# The Value of Intraoperative Frozen Section Analysis for Margin Status in Breast Conservation Surgery in a Nontertiary Institution

**DOI:** 10.1155/2014/715404

**Published:** 2014-09-30

**Authors:** Mona P. Tan, Nadya Y. Sitoh, Amanda S. Sim

**Affiliations:** MammoCare, 38 Irrawaddy Road, No. 06-21, Singapore 329563

## Abstract

*Introduction*. Breast conservation treatment (BCT) for early-stage breast malignancies requires negative margins and good cosmesis. Reoperations may be needed to achieve negative margins, which can have an adverse impact on outcomes. This study was performed to evaluate the efficacy of intraoperative frozen section analysis (IFSA) for margin assessment to reduce excision rates. *Methods*. All patients treated at the authors' private healthcare facility between 2009 and 2011 for breast cancer were included in the study. Those for whom BCT was intended underwent wide excision with IFSA. Six margins of the excised tissue, and the sentinel lymph node (SLN), where appropriate, were submitted for IFSA. Patient demographics, tumour characteristics, number of operations performed, and outcomes were analysed. *Results*. Of the 161 patients analysed, 138 (85.7%) had successful breast conservation. Four patients required a reoperation for incomplete surgical extirpation. One had a false negative SLN assessment on IFSA, and was returned to the operating room for an axillary dissection. Three patients required reoperations for inadvertently missed multicentric disease. None had false negative margin evaluation with IFSA necessitating reexcision. *Conclusion*. The use of IFSA allows low rates of reoperation with BCT. Further research is needed to establish consistency in low reexcision rates for cost-effectiveness and optimum resource allocation.

## 1. Introduction

Although therapeutic decisions have long been made on the basis that breast conservation treatment (BCT) offers equivalent survival outcomes compared to mastectomy for the treatment of breast cancer [1], a recent analysis suggesting that BCT is associated with higher breast cancer specific survival [2] may change that paradigm. This new data elevates and strengthens the position of BCT as the surgical procedure of choice. However, one area of concern for BCT is the potential need for multiple operations to achieve the requisite negative margins to ensure optimum local control. Multiple surgical episodes have several undesirable consequences [3], such as higher levels of patient anxiety, delays in adjuvant treatment, and possible poorer cosmetic outcomes. Therefore, there has been a call to devise methods to reduce reexcision rates [3]. Where there is no intraoperative assessment of margin status, rates of reoperation in general have been reported to be in excess of 20% [3–5]. Techniques to decrease reoperations include ultrasound assessment, cavity shave margins, touch preparation cytology, and intraoperative frozen section analysis (IFSA) [4–9]. Though being commonly used, the limitations of routine IFSA for margin status include time resource allocations, labour intensity, technical challenges, and cost considerations [10, 11]. There is data to suggest that a reduction in reexcision rates from 26% to 3% has been shown to be associated with a fiduciary benefit to both the provider and payor in the United States [5]. Hence, the objective of intraoperative margin assessment would be to lower reexcision rates to less than 3% for cost-effectiveness. This study was therefore conducted to investigate if the use of IFSA in a private hospital setting could achieve this goal of reducing rates of reoperation to below this stipulated level. Secondary objectives were to determine factors associated with reexcision and identify potential areas of improvement in terms of decreasing repeated operations for BCT.

## 2. Methods

**Figure 1:**
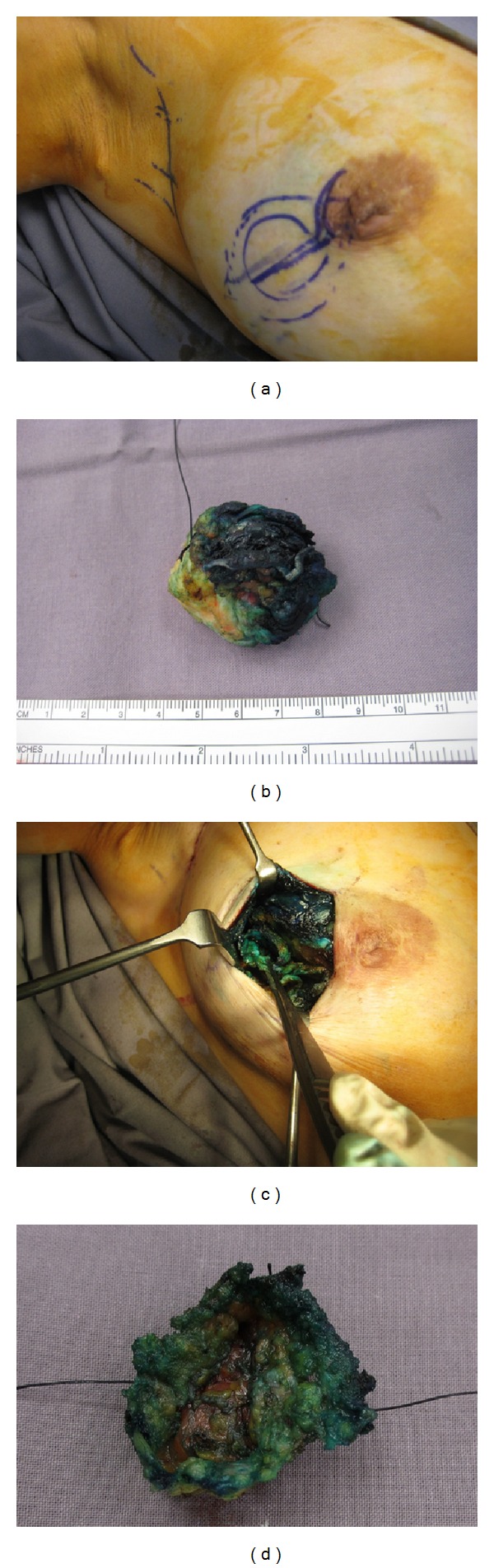
((a)–(d)) Sequential pictures of how a “saucer” margin is resected through a boomerang incision [12] with a lateral limb for this 17 mm grade 3 invasive ductal carcinoma sited in a periareolar position. Such a “saucer” margin offers best outcomes when performed in “virgin” tissue planes; hence, intraoperative frozen section analysis affords the pathologist an enhanced role in BCT.

A retrospective analysis of all patients with breast malignancies who underwent operative treatment at the authors' private medical facility between January 2009 and December 2011 was performed. Diagnosis of breast cancer was made based on preoperative pathological assessment and not on IFSA. Following diagnosis, patients deemed eligible for BCT were given the option of an attempt at breast conservation or mastectomy, with or without reconstruction. Those who had tumours assessed to be too large for BCT were referred to for neoadjuvant medical treatment if they wished for downstaging to attempt BCT. Women with impalpable lesions who chose BCT had preoperative image localisation. Patients intended for BCT underwent IFSA for margins and sentinel node evaluation where appropriate. Sentinel lymph node (SLN) biopsy was performed using blue dye injection only [13] and was performed prior to wide excision. The intraoperative objective was to obtain a gross circumferential margin of 10 mm with specimen orientation using sutures. IFSA for the SLN was done either for the whole node or after bisecting the node, depending on the size of the submitted node and on the pathologist's preference. The tissue allocated for analysis was then frozen in liquid nitrogen and sectioned. Six margins of the wide excision specimen were processed either by shaved margins or in a perpendicular fashion according to the pathologist's preference and then submitted for frozen section analysis. Margin positivity detected during surgery was treated by excision of further margins until negative margins were achieved. Where appropriate, a “saucer margin” was excised during the time taken for IFSA to be completed. This consisted of a circumferential margin of 3–5 mm thickness around the biopsy cavity in continuum with the base (Figures 1(a), 1(b), 1(c), and 1(d)). Frozen section analysis of this “saucer margin” was directed by the findings of the original tumour excision specimen. Only the relevant margins of the “saucer” were examined by IFSA. Local tissue rearrangement was then performed by mobilisation of full thickness parenchymal flaps and apposition of the tissue pillars with sutures. This was followed by raising short lengths of skin flaps prior to skin closure to prevent deformity and optimise cosmesis.

The final margin status was based on haematoxylin & eosin (H&E) staining with “no ink on tumour” defined as a negative margin. SLN status was confirmed with 2 or 4 serial sections at 6 microns on H&E. The number of surgical procedures required to obtain clear margins and complete axillary staging was noted. Patients were deemed to have successful BCT if they underwent breast conserving surgery with pathologic clear margins and completed all recommended adjuvant medical treatment, followed by radiotherapy. They were subsequently followed up for local and distant recurrence.

Statistical analyses were performed using SPSS (Chicago, IL) version 11 advanced statistical software module. Where appropriate, comparisons of categorical variables were performed using the chi-squared test and continuous variables with median or mean values were compared using the Mann-Whitney *U* test.

## 3. Results

A total of 163 patients underwent surgical treatment during the study period. Two patients did not complete recommended adjuvant therapy and were excluded, leaving 161 patients for analysis. All patients were female.  Table 1 records demographic, tumour characteristics, and surgical treatment modality of the study population.

Of the 161 patients, 139 were intended for breast conserving surgery, and 138 underwent successful BCT (85.7%). Of those for whom BCT was the initial intended surgical treatment, there were 4 women who required a second operation, and none had a third therapeutic operation. The reasons for the procedures are summarised in Table 2. Patient A had a false negative reading of IFSA on the SLN, prompting a second operation for axillary dissection. Three other patients had undetected multicentric lesions prior to their first surgery. Two patients (Patients B & C) presented with palpable lesions associated with microcalcifications occupying a significant proportion of the involved segment. Although the extent of the microcalcifications was bracketed for the primary operations, postoperative mammography demonstrated additional microcalcifications in a different segment. The second clusters of microcalcifications were superimposed on the initial larger area of calcifications. Patient B was elected for a completion mastectomy with reconstruction, and Patient C underwent a reexcision. Both patients are currently disease-free at 46 and 32 months after treatment, respectively. The last patient, Patient D, had an inadvertently missed lesion that was not visualised on standard preoperative imaging with mammography and sonography. She initially underwent wide excision of a palpable left breast tumour at the 9 o'clock position with clear margins. A positive SLN at IFSA led to a completion axillary dissection at her primary surgery. Postoperatively, a staging positron emission tomography (PET scan) demonstrated a suspicious 10 mm lesion at the 12 o'clock position in the ipsilateral breast, corroborated on a review ultrasound examination. As such, she underwent a second operative procedure consisting of ultrasound localisation with wide excision of the 12 o'clock tumour. Margins were negative on IFSA.

The mean pathologic tumour size for those requiring reoperation was 32.3 mm while that for those without a second therapeutic procedure was 21.3 mm (*P* = 0.17). There was no significant difference in the requirement for a second procedure based on histologic type (*P* = 0.87), whether invasive or in situ disease, neither was there reexcision performed for patients who underwent neoadjuvant chemotherapy followed by BCT.

There were no patients who required a reexcision solely on the basis of a false negative IFSA of margins. In other words, a negative margin status on frozen section was confirmed on permanent paraffin sections for all patients who underwent BCT. After a median follow-up period of 45 months, local control for women who underwent BCT was 98.4%. All who had a reoperation are currently disease-free. There was no difference in local control or distant events between women who had one or more therapeutic procedures (*P* = 0.93). These findings are summarised in Table 3.

## 4. Discussion

Successful BCT requires the extirpation of tumour with negative surgical margins, preserving sufficient tissue volume for good cosmesis. Although BCT is considered a standard of care for the treatment of early breast cancer, concerns have been raised regarding the need for multiple therapeutic procedures for positive margins. Reoperation rates as high as 72% [15, 16] have been reported, which can result in patient dissatisfaction [3]. With recent data encouraging the use of BCT [2], the issue of reexcisions certainly needs to be addressed. A reduction of reexcision rates from above 26% to 3% or below with IFSA has been shown to be associated with a treatment cost benefit [5]. This cost-effectiveness is in addition to the expeditious therapeutic course the patient will undergo with minimal surgical interventions.

Dr. Jorns et al. reported a relatively high reexcision rate, attributing this to an increased willingness to attempt BCT in more complex cases [10, 14]. Her group found that IFSA reduced reoperations from 48.6% to 14.9%, a decrease largely due to margin assessment and not SLN [10]. The BCT rate at that centre was reported to be 63% in a separate study [14]. In the present study, overall BCT rate was higher at 85.7%, with a reexcision rate of 0.8% for a falsely negative IFSA assessment of the SLN. There were no reexcisions for margins. With a total reoperation rate of 2.5%, our data suggests that a sufficiently low reexcision rate for cost-effective treatment in the presence of a high BCT rate is possible with routine IFSA [5].

In order to optimise the efficacy of IFSA, margins should be assessed in conjunction with SLN. If only the SLN were assessed, the reduction in reoperations was reported to be 7% [17]. A similar dilemma would be expected if only margins were reviewed intraoperatively and SLN status was determined histologically. Therefore, it is necessary to perform IFSA on both margins and SLN to avoid difficulties encountered elsewhere [17]. Even with the use of IFSA, a definition of negative margin is necessary, and “no ink on tumour” was used in this cohort for determining the need for additional shave margins [18, 19].

Apart from IFSA, other techniques for margin assessment have also been studied. These include intraoperative ultrasound, digital specimen radiography, routine cavity shaved margins, and imprint cytology, as well as experimental techniques like radiofrequency spectroscopy and optical coherence tomography [4, 8]. Studies with IFSA consistently reported high accuracy rates but were found to add 20–30 min on operating time. The additional time waiting for the pathologist's assessment could be used by the surgeon to excise a “saucer margin.” As described earlier, this entails excision of a circumferential margin of between 3 and 5 mm thickness around the tumour cavity together with the base, akin to cavity shaved margins removed as a continuous tissue segment. This procedure is more efficiently performed at the time of the primary operation through “virgin planes,” rather than through granulation tissue as in a reexcision, for the latter tends to be friable and may require tissue excision of greater thickness for both control during dissection and adequate pathologic assessment. Avoiding excessive tissue loss is particularly important for women with smaller volume breast tissue where volume of retained breast parenchyma needs to be optimised for cosmesis. This dual form of intraoperative management used in our study, combining IFSA and “saucer” margins, may have contributed to low reexcision rates.

Opponents to IFSA suggest that, rather than its routine use, the means of reducing the need for reexcision should be centred on the use of pathologic and molecular prognostic factors to determine indications for reoperations [18]. This approach is expected to lower reexcision rates and costs of treatment [18, 19]. However, with positive margins reported to average 44% [7, 17], there is yet no evidence at this time to show that the use of a policy of “no ink on tumour” alone could consistently lower reexcision rates to a level below the threshold necessary to negate cost-effectiveness of IFSA [5]. In the private healthcare facility in Singapore where the study was conducted, the cost of hospital processes for a reoperation is expected to be between $2,675 and $3,645, while the cost of IFSA is between $457 and $985. For the individual patient undergoing surgery in this facility, it would be logical to use IFSA as a cost-effective approach to avoid a reexcision.

Ductal carcinoma in situ and invasive lobular subtype have been shown to be associated with an increased risk for positive margins and reexcision [9, 18]. However, this was not evident in our series. A possible reason may be the relatively small cohort size. Another plausible explanation may be related to the routine use of IFSA with frequent cooperation and communication between surgeon and pathologist, resulting in additional shave margins taken from the appropriate sites at the point of the primary surgery. Such technical nuances facilitate single-stage procedures [20].

While none of the study patients required reexcision for a false negative result on IFSA, three needed reoperations for inadvertently missed multicentric lesions. Although routine preoperative MRI may have avoided this, its routine use increases the odds of having a mastectomy without significant reduction in reexcision rates or mortality [21]. A selective approach may be more appropriate, but data is lacking on the indications for preoperative MRI that enable reduced reexcision rates without increased mastectomy rates. Three patients in this study are with inadvertently missed multicentric tumours, of whom two had lesions in excess of 50 mm associated with microcalcifications. The latter finding could serve as an indication for preoperative breast MRI but further research is required to verify this.

The small cohort size serves as a limitation in this study and may explain its unexpectedly encouraging results. The false negative rate for IFSA for both margins and SLN was 0.8%. To the authors' knowledge, only one other group reported a similar false negative rate for frozen section in breast lesions, though in slightly different circumstances [22]. This low false negative rate may be due to a routine use of IFSA in this setting to decrease multiple operations and costs for the patient, thereby increasing pathologists' experience with the technique and resultant accuracy [23]. Admittedly, it would be challenging to reproduce such low reexcision rates and it is acknowledged that further research is needed to evaluate reproducibility of high accuracy rates for IFSA. Notwithstanding these limitations, the data contributes to the growing body of evidence on the efficacy of IFSA in decreasing reexcision rates and serves as a reference point for future work. In conjunction with data from larger tertiary institutions [24, 25], the information could assist in the implementation of a practical clinical approach to allow the benefits of IFSA to be offered across a broad range of healthcare settings.

## 5. Conclusion

In the presence of a BCT rate of 85.7% in this study cohort, 2.5% of patients underwent reoperations. None had reexcision for falsely negative margins at IFSA, nor did any patient require a third therapeutic operation. A low rate of reexcision is possible using IFSA for BCT in this nontertiary private healthcare facility, with acceptable short-term local control. This data is comparable to other contemporary series from larger institutions (Table 4). With the future possibility that BCT will be considered superior to mastectomy in terms of breast cancer specific survival [2], it is increasingly important to streamline care for its optimum efficiency and efficacy and to ensure homogeneity in the care of women with breast cancer [3]. Further investigations are needed on the applicability of IFSA across a broad range of healthcare settings to avoid wide discrepancies in surgical reexcision rates.

## Figures and Tables

**Table 1:** Summary of demographic, clinicopathologic, and outcome data for study population.

Clinicopathologic characteristic	All patients {*n* = 161} (%)		BCT {*n* = 138} (%)	Mastectomy {*n* = 45} (%)	*P* value
Age in years					
Median (range)	48 (28–78)				
Mean (SD)	48.8 (9.8)		48.3 (10)	52.4 (8.3)	**0.04**
Ethnicity					0.87
Chinese	106	(65.8)	91	15	
Malay/Indonesian	11	(6.8)	9	2	
Indian	12	(7.5)	11	1	
Other Asian	14	(8.7)	13	1	
Caucasian	18	(11.2)	14	4	
Mode of presentation					**0.02**
Symptomatic tumours	116	(72)	95	21	
Screen detected lesions	45	(28)	43	2	
All patients	161		138 (85.7%)	23 (14.3%)
				By need 15 (9.3%)
				By choice 8 (5.0%)
Tumour size in mm (range)					
Median (range)	19.0 (4–97)				
Mean (SD)	21.6		19.7 (12.4)	33.0 (26.0)	**<0.001**
T1	100	(62.1)	92	8	
T2	50	(31.1)	39	11	
T3	8	(5.0)	5	3	
T4	3	(1.8)	2	1	
Stage at diagnosis					**<0.001**
0	20	(12.4)	18	2	
I	67	(41.6)	66	1	
II	55	(34.2)	46	9	
III	18	(11.2)	8	10	
IV	1	(0.6)	0	1	
Histological type					0.39
DCIS	20	(12.4)	18	2	
Invasive ductal	125	(77.6)	108	17	
Invasive lobular	7	(4.4)	5	2	
Other invasive	9	(5.6)	7	2	
Neoadjuvant medical therapy					
Yes	23	(14.3)	14	9	
No	138	(85.7)	124	7	
Disease extent					0.06
Unifocal	121	(75.2)	104	17	
Multiple foci at diagnosis	40	(24.8)	34	4	
Recurrence					0.26
Local recurrence	3	(1.9)	2 (1.4%)	1 (4.3%)	
Distant disease/death	4	(2.5)	2 (1.4%)	2 (8.7%)	
*Median follow-up (months) *	*45 *				
*(range) *	*(18*–*64) *				

BCT: breast conservation surgery; SD: standard deviation.

DCIS: ductal carcinoma in situ.

**Table 2:** Summary list of the repeat procedures for incomplete primary surgical treatment of cancer for this cohort and the attendant reasons.

Patient	Repeat operation	Reason
A	Completion axillary dissection	Macrometastasis detected on histology, not visualised on IFSA

B	Completion mastectomy	Multicentric disease with microcalcifications superimposed, not visualised prior to primary operation, margins clear at first surgery

C	Re-excision of multicentric disease	Multicentric disease with microcalcifications superimposed, not visualised prior to primary operation, margins clear at first surgery

D	Re-excision of multicentric disease	Multicentric disease, consisting of two mass lesions: the first palpable tumour was the presenting symptom, and the second impalpable lesion was undetected prior to first operation. Following identification through a PET Scan, she underwent a reoperation through the same incision

	Re-excision for margins	None

**Table 3:** Summary of demographic, clinicopathologic, and outcome data comparing those patients with and without reoperation.

Clinicopathologic characteristic	All patients {*n* = 161} (%)		No reoperation {*n* = 157}	Reoperation {*n* = 4}	*P* value
Age in years					
Median (range)	48 (28–78)				
Mean (SD)	48.8 (9.8)		48.9 (9.9)	48.4 (8.6)	0.91
Mode of presentation					
Symptomatic tumours	116	(72)	112	4	0.21
Screen detected lesions	45	(28)	45	0	
Tumour size in mm (range)					
Median (range)	19.0 (4–97)				
Mean (SD)	21.6		19.3 (15.6)	33.3 (16.8)	0.17
Stage at diagnosis					0.32
0	20	(12.4)	19	1	
I	67	(41.6)	67		
II	55	(34.2)	52	3	
III	18	(11.2)	18		
IV	1	(0.6)	1		
Histological type					0.87
DCIS			19	1	
Invasive disease only			56	1	
Invasive disease with DCIS			80	2	
Other invasive			2	0	
Neoadjuvant medical therapy					
Yes	23	(14.3)	14	23	0.41
No	138	(85.7)	134	4	
Disease extent					**0.04**
Unifocal	121	(75.2)	120	1	
Multiple foci at diagnosis	40	(24.8)	37	3	
Recurrence					0.95
Local recurrence	3	(1.9)	2 (1.4%)	0	
Distant disease/death	4	(2.5)	2 (1.4%)	0	
*Median follow-up (months) *	*45 *				
*(range) *	*(18*–*63) *				

SD: standard deviation.

DCIS: ductal carcinoma in situ.

**Table 4:** A summary comparison of published data.

Author	Findings on reexcision rate (BCT) using IFSA for margins	Other relevant findings/comments
Fukamachi et al. [6]	Reduction of margin positive rates from 27% to 9.8%	

Esbona et al. [9]	Reexcision rates decreased from 27% to 6%	Systematic review

Jorns et al. [10]	Reexcision rates decreased from 48.6% to 14.9%	Reoperation rates decreased from 55.3% to 19.3% for BCT rates of 63% [14]

**Current study**	**0%** **No reexcision rates for margins with IFSA**	**Reoperation rates for axillary node positivity 0.8% for BCT rates 85.7%**
